# Armenian or Diamond Cement

**Published:** 1866-10

**Authors:** 


*Armenian or Diamond Cement.*—This article, so much esteemed for uniting pieces of broken glass, for repairing precious stones, and for cementing them to watch-cases and other ornaments, is made by soaking isinglass in water until it becomes quite soft, and then mixing it with spirit in which a little gum mastic and ammoniacum have been dissolved. The jewelers of Turkey, who are mostly Armenians, have a singular method of ornamenting watch-cases, etc., with diamonds and other precious stones, by simply glueing or cementing them on. The stone is set in silver or gold, and the lower part of the metal made flat, or to correspond with the part to which it is to be fixed; it is then warmed gently, and has the glue applied, which is so very strong that the parts so cemented never separate. This glue, which will strongly unite bits of glass, and even polished steel, and may be applied to a variety of useful purposes, is thus made in Turkey: Dissolve five or six bits of gum mastic, each the size of a large pea, in as much spirits of wine as will suffice to render it liquid; and in another vessel dissolve as much isinglass, previously a little softened in water, (though none of the water must be used,) in French brandy or good rum, as will make a two ounce phial of very strong glue, adding two small bits of gum albanum, or ammoniacum, which must be rubbed or ground till they are dissolved. Then mix the whole with a sufficient heat. Keep the glue in a phial closely stopped, and when it is used, set the phial in boiling water. Some persons have sold a composition under the name of Armenian cement in England; but this composition is badly made; it is much too thin, and the quantity of mastic is much too small. The following are good proportions: Isinglass, soaked in water and dissolved in water, (thick;) dissolve in this ten grains of very pale gum ammoniac, (in tears,) by rubbing them together; then add six large tears of gum mastic, dissolved in the least possible quantity of rectified spirits. Isinglass, dissolved in proof spirit, as above, three ounces; bottoms of mastic varnish, (thick but clear,) one and a half ounces; mix well. When carefully made, this cement resists moisture and dries colorless. As usually met with, it is not only of a very bad quality, but sold at exorbitant prices.—*Tinman's Manual and Scientific American.*

---

*Bromide of Potassium in Sleeplessness consequent upon Uterine Irritation.*—It is better always to try this agent before resorting to narcotics in the wakefulness which is often associated with disease of the uterus or its appendages. Indeed, in some instances it will succeed in inducing rest where the others fail, and beside, the liability of these to disturb the digestive organs, is sometimes a serious objection to their administration. Without detailing cases, I would simply state that I have recently found remarkable benefit in the condition mentioned, from the bromide of potassium—the dose should hardly be less than ten grains, and may be twenty, or more.—*Cincinnati Journal of Medicine.*

---

*Decision against River Pollution.*—A case which has been twenty-five years in dependence, has recently been decided, after a trial of eleven days, in Edinburgh, Scotland. The paper makers beside the river North Esk have been polluting that stream with the refuse of their business until the landed gentry could no longer endure it, so they pushed the case to a decision and obtained a verdict prohibiting such use of the water course. Citizens of Chicago would be delighted to obtain such a verdict against those who have converted their would-be river into a cesspool. But large bodies move slowly.

